# Perspectives on a mobile application that maps assistive technology resources in Africa

**DOI:** 10.4102/ajod.v8i0.567

**Published:** 2019-08-22

**Authors:** Surona Visagie, Rebecca Matter, George Kayange, Mussa Chiwaula, Mark Harniss, Callista Kahonde

**Affiliations:** 1 Centre for Rehabilitation Studies, Stellenbosch University, Cape Town, South Africa; 2 School of Public Health and Family Medicine, University of Cape Town, Cape Town, South Africa; 3 Southern Africa Federation of the Disabled (SAFOD), Gaborone, Botswana; 4 Department of Rehabilitation Medicine, University of Washington, Washington, United States

**Keywords:** assistive products, assistive technology, disability, mobile application, low resourced settings

## Abstract

**Background:**

Access to assistive technology (AT) is poor in African countries because of a lack of knowledge, resources, services and products. A mobile application (app), the AT-Info-Map, was developed to map AT availability in southern Africa.

**Objectives:**

This article aimed to describe users’ and suppliers’ perceptions of the AT-Info-Map app.

**Method:**

Qualitative data were collected in Zambia, Botswana, Malawi and Lesotho, through nine focus group discussions with 72 participants. Participants included AT users, AT suppliers and representatives of disability organisations. Data were thematically analysed.

**Results:**

Two broad themes, that is, *usefulness of the AT-Info-Map application* and *technical issues and content*, emerged from the data analysis. Subthemes under usefulness focused on the importance of using current technology, convenience of the app, the need for accuracy, responsiveness of supplier to user’s needs, influence on AT market and how the app creates an opportunity for networking. Challenges to download and navigate the app, the need for training in its use, exclusion of those not literate in English and those with visual impairments were subthemes under technical issues and content.

**Conclusion:**

The app was perceived as an important step to increase access to AT for persons with disabilities in less resourced settings. The challenges that emerged from the data analysis have led to the development of a web-based system that will complement or replace the app and improve AT information provision. However, the information provided by the app and website is still only a partial solution to improve AT access in Southern Africa.

## Introduction

Assistive technology (AT) is defined as:

any item, piece of equipment or product, whether it is acquired commercially, modified or customized, that is used to increase, maintain, or improve the functional capabilities of individuals with disabilities. (World Health Organization [WHO] 2011:101)

Assistive technology can compensate for loss of function caused by various impairments, for example sensory deficits, cognitive impairment and paralysis. It can enhance participation in social roles and ultimately quality of life (WHO 2018). As shown by Tebbutt et al. (2016), AT has a crucial role to play in ensuring that persons with disabilities are not left behind in the quest to achieve the sustainable development goals. Internationally, the WHO is placing a strong focus on the provision of appropriate AT services and products through the Global Cooperation on Assistive Technology (GATE) initiative (WHO 2018) and the inclusion of AT in the Rehabilitation in Health Systems document (WHO 2017).

However, the majority of people from developing countries who need AT do not have access to it (Matter et al. 2017). For those who do have access, the AT is often not a lasting solution because of the lack of repair and replacement services (Visagie et al. 2016). The need for AT in Africa is also on the increase as population aging occurs (Garçon et al. 2016), non-communicable diseases increase in prevalence (Mayosi et al. 2012), people live longer with the sequela of HIV (Brothers et al. 2014) and trauma is still ever present (Mayosi et al. 2012). In Africa, specifically, a myriad of challenges related to a lack of funding, knowledge and awareness, resources, services and products hamper access to AT (Harniss, Raja & Matter 2015; Matter et al. 2017; Visagie et al. 2016).

mHealth, ‘the use of mobile technologies for health related activities’, is rapidly expanding throughout the world (Vesel et al. 2015:1684). Service provision and management of complex conditions, such as non-communicable diseases (Opoku, Stephani & Quentin 2017) and palliative care (Allsop, Namisango & Powell 2018), benefited from mHealth applications in Africa. Mobile phone applications or mHealth applications have the advantage of reaching large populations in different geographical areas across gender and social divides (Vesel et al. 2015). It connects users to services that were previously unavailable to them or were only available through substantial effort in terms of travel, expenses and waiting times (Abaza & Marschollek 2017; Opoku et al. 2017; Vesel et al. 2015; Watkins et al. 2018).

mHealth applications can have various purposes, including self-diagnosis and diagnostic support (Abaza & Marschollek 2017; Vesel et al. 2015), marketing of services (Vesel et al. 2015), exercises, education and counselling (Abaza & Marschollek 2017; Allsop et al. 2018; Vesel et al. 2015), health status monitoring (Abaza & Marschollek 2017) and adherence to management regimes (Abaza & Marschollek 2017; Allsop et al. 2018; Vesel et al. 2015). Further applications include emergency medical response (Abaza & Marschollek 2017; Vesel et al. 2015), drug supply chain monitoring (Vesel et al. 2015), transfer of funds (Vesel et al. 2015), maintaining medical databases (Abaza & Marschollek 2017; Allsop et al. 2018; Vesel et al. 2015) and consultations between healthcare providers (Abaza & Marschollek 2017; Allsop et al. 2018; Opoku et al. 2017; Vesel et al. 2015; Watkins et al. 2018).

Even so, mHealth does face a number of challenges. This includes network fees (Allsop et al. 2018; Vesel et al. 2015; Watkins et al. 2018), low literacy levels of users (Krah & de Kruijf 2016; Watkins et al. 2018), lack of evidence of its long-term impact on health indicators (Opoku et al. 2017; Vesel et al. 2015), poor infrastructure (low ownership of smart phones and poor Internet access) in developing countries (Allsop et al. 2018; Vesel et al. 2015; Watkins et al. 2018), privacy (Allsop et al. 2018; Krah & de Kruijf 2016; Vesel et al. 2015; Watkins et al. 2018), security (Allsop et al. 2018; Vesel et al. 2015) and challenges with interoperability between systems (Vesel et al. 2015). In addition, sustainability of programmes was also flagged as a challenge. This was especially true for programmes that were initiated through external donor funding without involvement of government in developing countries (Allsop et al. 2018; Vesel et al. 2015).

In 2016, the Southern African Federation for the Disabled (SAFOD) in conjunction with partners from the University of Washington (USA), Stellenbosch University (South Africa) and Dimagi (https://www.dimagi.com/) received funding from the Google Impact Challenge: Disabilities (https://www.google.org/impactchallenge/disabilities/) to develop an mHealth smart phone application (app) to map AT availability in southern Africa. The purpose of the AT-Info-Map app was to assist users and providers of AT in locating sources of AT such as private companies and non-governmental organisations (NGOs), and to show gaps in the availability of AT in southern Africa (Visagie et al. 2018). The app had the capacity to be used offline once installed, an advantage in settings, such as southern Africa, where resources and infrastructure are limited (Allsop et al. 2018; Vesel et al. 2015; Watkins et al. 2018). Scoping and design, led by Dimagi (https://www.dimagi.com/), found that data in the app should include information about AT suppliers and disability service organisations (Visagie et al. 2018). The app was first piloted and refined in Botswana in 2016 and early 2017 (Visagie et al. 2018). After refinement the app was implemented in four other countries in 2017 (Zambia, South Africa, Malawi and Lesotho). Implementation in the last five SAFOD countries (Angola, Mozambique, Namibia, Swaziland and Zimbabwe) started in April 2018. Implementation involved hiring and training local administrators within each country, who then identified and entered data about AT suppliers and disability service providers. The Southern African Federation for the Disabled provided training and monitoring of country-level data entry by administrators. Data collection and validation are ongoing to ensure that AT supplier and service information is accurate and coverage is comprehensive in all 10 countries.

Lack of monitoring and evaluation was flagged as a major issue in many mHealth programmes (Vesel et al. 2015). To partially address this challenge within the current programme (AT-Info-Map), user feedback was sought through focus group discussions. This article aims to describe perceptions of the use of the AT-Info-Map app through information that was gathered from key AT stakeholders during routine monitoring after rollout in the first five countries: Botswana, Zambia, South Africa, Malawi and Lesotho.

## Methods

Data collection and analysis were done according to a qualitative descriptive design. Data were collected using nine focus group discussions in the five southern African countries where the app has been implemented in 2016–2017, that is, Zambia, Botswana, Malawi, South Africa and Lesotho. Data from the South African focus group had to be excluded as challenges were experienced with collaboration with SAFOD representors in that country that resulted in insufficient information on the data collection process. (No records were kept of the focus group participants’ demographic details, and quotes were not linked to individual participants in the transcript.)

Focus group participants were conveniently selected from those who received training in the use of the app during the implementation of the AT-Info-Map in each country. The Southern African Federation for the Disabled and their affiliates within each of the four countries identified and recruited AT stakeholders to participate in the implementation trainings, totalling 352 individuals (Zambia: 112, Botswana: 67, Malawi: 86, Lesotho: 87). Assistive technology stakeholders included people with disabilities who used AT, suppliers of AT and representatives from NGOs that advocate for and/or serve people with disabilities.

Data were collected in English as that is the official medium of communication in all five countries and the language used in the app. The focus group discussions were guided by a schedule developed by the programme’s leadership team. The schedule included the following main concepts:

- Feasibility of the app (usefulness, challenges, benefits, accuracy and completeness).
- Technical issues (downloading and installation, instructions, data access).
- Possible outcomes of the app (awareness, AT demand, AT supply).
- General dis/satisfaction with the app.
- Suggestions for change.
- General AT information (awareness and knowledge, policy implementation, supply chain, procurement and provision, opportunities, interventions).

The Southern African Federation for the Disabled took the lead in the monitoring process and employed and trained focus group discussion facilitators, who were members of SAFOD.

Focus group discussions were audio recorded and transcribed by a member of SAFOD. Inductive thematic analysis was done by the first author. She familiarised herself with the data through multiple readings of printed copies of the transcriptions. Through notes in the margins she started to identify like codes. These codes were then organised into meaningful groups that became subthemes or an overarching theme that encompasses various subthemes. These themes were reviewed by co-authors to ensure clarity, and were then finalised, defined and named (Braun & Clarke 2006).

### Trustworthiness

Convenient sampling negatively impacted credibility of the findings. However, the large number and heterogeneousness of participants should offset this and enhance credibility. In addition, data saturation was achieved. Furthermore, both positive and negative findings were presented. Confirmability is strengthened by basing themes on the perceptions of participants and supporting them with narrative examples as done in this article. The article provides information on the methods used and should allow other researchers to replicate the study. The information on methods and the setting should also guide an informed decision on whether findings can be transferred to other similar settings (Given 2008).

### Ethical considerations

The monitoring process received ethics approval through University of Washington Human Subjects Division (HSD) (STUDY00003701). There were no risks to participants. Participation in the focus groups was voluntary in nature, and verbal informed consent was obtained. This included consent to the use of an audio recording device. Participants of individual focus groups entered into a group contract. All information is treated as confidential, was anonymised and encrypted data storage was used. Data were stored on password-protected computers. During dissemination no identifying particulars will be used. Participants were reimbursed for travel expenses, and refreshments were provided.

## Results

### Participants

Nine focus group discussions took place in the four countries from December 2017 to January 2018, including 72 participants in total (see Table 1).

**TABLE 1:** Characteristics of focus group participants per country.

Country	Total (*N*)	No. of focus groups	Women	AT users	AT suppliers	NGO representatives†	Other‡
Zambia	20	2	6	7	7	12	4
Botswana	11	2	4	7	6	4	-
Malawi	19	2	5	10	3	2	4
Lesotho	22	3	8	17	-	5	5

**Total**	**72**	**9**	**23**	**41**	**16**	**23**	**13**

AT, assistive technology; NGO, non-governmental organisation.

† , In some instances, persons with disabilities also represented NGOs or suppliers; therefore, the numbers in columns 5–8 add to more than the total number of participants.

‡ , Other includes mainly caregivers.

Table 2 shows that focus group participants had experience with a wide range of AT types, both as users of AT and organisations or companies that supply AT. The majority of participants (*N* = 29) had experience with AT for physical or mobility impairments.

**TABLE 2:** Background information related to impairments and assistive technology experience of participants.

Variable	Users	Suppliers	Service providers	Caregiver or assistant	NGOs	Other
Physical or mobility impairments	20	5	-	-	4	-
Visual impairments	4	3	-	1	3	-
Hearing impairments	3	-	-	3	-	-
Mental impairments	1	-	-	-	3	-
Intellectual impairments	1	-	-	-	-	-
Impairment type unknown or general	12	4	5	3	13	6

NGO, non-governmental organisation.

### Themes

Data were analysed across the different focus groups and are presented across groups and countries. Opposing opinions are brought to the fore, and suggestions for improvement are presented in an integrated fashion as relevant to each subtheme. Two broad themes emerged from the data:

- Usefulness of the AT-Info-Map application
- Technical issues and content

Subthemes were identified through an inductive process under each of the two main themes and are presented in Table 3.

**TABLE 3:** Themes and subthemes from the data.

Themes	Subthemes
Usefulness of AT-Info-Map application	Current technology
	Convenience
	Accuracy and completeness
	Responsiveness of suppliers
	Growth in the AT market
	Networking
Technical issues and content	Download and use
	Icons and navigation
	Training
	Terminology
	Not fully inclusive
	Hardware and data

AT, assistive technology.

#### Theme I: Usefulness of the AT-Info-Map application

Focus group participants from all four countries were enthusiastic about the app. Provision of information for persons with disabilities, who are often excluded or marginalised, through the use of *current technology* was seen as a progressive step.

‘I think the coming of this app is quite revolutionary because in the past we had no digital platform where suppliers of various ATs and devices can advertise and display their products and services.’ (Participant 1, Zambia, Man, Supplier of AT for vision)

‘…it’s quite amazing that even here in Botswana people are up to date with modern technology.’ (Participant 1, Botswana, Man, Physical disability)

The provision of information on different AT all on a single platform on your phone was seen as very *convenient*.

‘For persons with disabilities, movement and transportation is a challenge, the app will minimize their movement while they have access to information on their phones. This will be convenient, to call service providers and suppliers and ask them directly what you are looking for.’ (Participant 11, Botswana, Man, Albinism)

Having all products on a single platform will save time and money because it reduces the need to travel.

‘This will ease mobility and reduce time wastage in search for suppliers and services in as far as helping people with disabilities is concerned.’ (Participant 17, Malawi, Woman, NGO representing children with disabilities)

‘…it will help me in getting quotations easily without having to go to suppliers one by one. I will just call and enquire and it will reduce the cost associated with travelling from one supplier to supplier.’ (Participant 3, Botswana; Man, Physical disability)

In this regard, the information on location of suppliers is valuable as that shows the user where devices can be secured closest to him or her.

‘…it also makes it easier to locate and determine the actual distance between where the person is and where service provider is. So I think that part particularly is great innovation.’ (Participant 4, Zambia, Man, Supplier of mobility devices)

Participants from Lesotho had challenges in identifying locations on the app because it is not a common practice in Lesotho to name streets.

‘The challenge we face is the unknown streets in Lesotho, most streets are not seen within the country [*on the app*].’ (Participant 14, Lesotho, Man, Physical disability)

Participants were adamant that the information provided must be *accurate and complete*, a quality that some participants felt the app showed.

‘I believe it’s accurate because earlier on we noticed the Cheshire Foundation Botswana and Ambrose Academy they are just nearby and the location that they were showing they are approximately correct…even that ones that are outside Botswana the kilometers that we are getting here are exact.’ (Participant 9, Botswana, Man, Physical disability)

However, a participant from Lesotho differed.

‘There are many companies that are left behind in Lesotho. I have realized that organisations and institutions that I already know are not in the app.’ (Participant 11, Lesotho, Man, Physical disability)

Participants emphasised the importance of regular updating of the app to keep information current. They felt that dated information will quickly limit the use and positive experience of the app.

‘Sometimes you may lose trust in using them for example if you search for a product which you wanted you find it there and then you go to the suppliers you find that they don’t have it you might lose interest of using the application because you will be thinking that even the next time when you pick a product when you go there you won’t find it.’ (Participant 15, Zambia, Woman, Physical disability)

Participants felt that another advantage of the app is that it might *improve the responsiveness of suppliers* as it may facilitate opportunities for suppliers to receive feedback from the users. Suppliers thought that through the app they will become more sensitive to demands and what specific devices are needed and that they can use this information to provide more appropriate services.

‘On the supplier side, the app I think will be able to provide information on the demand for different products so that the supplier can be able to position themselves in a way that whenever the products are needed the supplier will be able to meet the demand.’ (Participant 8, Zambia, Man, Supplier of mobility devices)

The app might also assist with sourcing components and thus enhance supply.

‘It will really help us to be able to access information on the mapping from other stakeholders…you find that certain components will not be found here in Zambia but you can get these components maybe in the nearest countries. But in the previous days, we used to fly all the way from Zambia to Kenya just to go and get caster wheels. But as it is I’ve just seen from the information which has just been provided that almost the whole southern region here is covered.’ (Participant 6, Zambia, Man, Supplier of mobility devices)

The app will allow travellers with disabilities to source services as needed.

‘As the app is in Southern African countries, this gives people with disabilities travelling to these countries a much greater access to information of where they can find services and supplies when they need it.’ (Participant 8, Botswana, Woman, Health promotion and education provider)

Many participants thought the app will facilitate *growth in the AT market* through increasing awareness on available AT and the demand for AT.

‘As more people learn more about the app and start using it, finding information about different kinds of AT, the demand of AT will increase.’ (Participant 9, Botswana, Man, Physical disability)

‘For the suppliers, it will increase the market share…once your market share has increased, meaning even your income has to increase. Again, once your products are selling like hot cakes, there is a likelihood that you have to expand for instance; you have to expand to other provinces or districts…even increase employment opportunities in our respective organisations or companies.’ (Participant 5, Zambia, Man, Supplier of mobility devices)

The app *creates a platform for networking* between suppliers and users as well as suppliers with each other.

‘The app will bring organisations, suppliers and service providers together and this will make it easier for a local Malawian.’ (Participant 18, Malawi, Man, Supplier of AT)

‘I think this platform will also help service providers to be also interacting in between themselves. That will also help national development because they will be exchanging ideas of which I’m sure more services will be coming in and more other devices which may be ordered or made available to our service beneficiaries.’ (Participant 9, Zambia, Man, Supplier of AT for vision)

‘It’s going to benefit our organization in so many ways. Through this app, there is a possibility that we can identify other organizations outside the country that work on raising awareness and chances of networking and collaborating. The app basically is a platform to find networks and partners.’ (Participant 9, Botswana, Man, Physical disability)

Users made some suggestions for improvements to the app. The first was the inclusion of a toll-free phone number with other supplier information.

‘Also those providers if they can provide us maybe with a toll free line… because if we have a toll free line it will be easy for us to access this even if you don’t have talk time.’ (Participant 11, Zambia, Woman, Physical disability)

Participants also felt information could be enhanced through the addition of approximate prices and pictures.

‘What is important there is that they need to indicate the approximate prices so that you can also start budgeting before you even go there or you start even calling them.’ (Participant 17, Zambia, Man, Sensory disability)

‘I think they need to work around on the interface of the application, I like the icons they are quite simple and to the point, but maybe if they could add a little colour to the interface.’ (Participant 18, Zambia, Man, Caregiver of person with physical disability)

A search function that will show all services in a specific country was suggested.

‘So it would be far much better if these organizations can be categorized in countries e.g. Botswana, Zambia, etc. Another point is that, when maybe I want to go to Zambia for a visit, and I want to access the orthopedic and prosthetics services, how will I know if the orthopedic and prosthetics services information is from Zambia because they are not categorized according to country.’ (Participant 9, Botswana, Man, Physical disability)

#### Theme II: Technical issues and content

Technical issues caused major challenges. Some participants felt the app was easy to *download and install*.

‘It’s easily installed and easily accessible despite the fact that it asks some usernames and password to be use but those is very short usernames and password… it’s just easy password, all in all, easily installed, easily accessed and it’s not complicated, you just get what you want easily.’ (Participant 9, Botswana, Man, Physical disability)

However, many had trouble with this.

‘I failed to install the app easily especially the code.’ (Participant 11, Lesotho, Man Physical disability)

Participants questioned the need for a *password*.

‘After downloading the app, it needs a code and a password to be running. This is a challenge since many people are not well at remembering everything. These two should be removed for easy access.’ (Participant 16, Malawi, Man, NGO)

‘The main challenge is the installation codes and username and passwords that are already preconfigured. Though the username and password are easy, simple and quick to remember it will be difficult to encourage others to download and install the app as it will require the installation codes which are not accessible on Google Playstore. This will discourage more people to download and install the app.’ (Participant 8, Botswana, Woman, Health promotion and education provider)

Once downloaded some participants felt the *icons and navigation* were intuitive.

‘The instructions are very clear, very clear indeed. The app itself is quite easy to use. Everything is quite clear, for as long as somebody is literate. You just follow instructions.’ (Participant 1, Zambia, Man, Supplier of AT for vision)

However, not all participants could navigate and use the app without challenges.

‘After that [*remembering the password*] now the problem again is to review where you can enter the information. That’s the challenge which I’m facing because I tried last week, the whole week I haven’t got anything up to now, then the system tells me its corrupt.’ (Participant 4, Zambia, Man, Supplier of mobility devices)

‘The only challenge is that the app is advanced and needs people who are highly literate who have the technical knowhow on how phone applications work.’ (Participant 1 Malawi, Man, Journalist focussing on disability issues)

Participants agreed that *training* on the app should continue.

‘We need frequent trainings on the app, and almost the whole country should be reached by such trainings.’ (Participant 12, Malawi, Women, Student who used the app)

Participants felt persons with disabilities have an important role to play in ensuring that the app is useful and sustainable.

‘The way it can be sustainable is to engage more DPOs to take part in the project. For the app to be able to be sustained, there is need to be an awareness and education regarding AT. When embarking on the awareness phase, collaborating and including people with disabilities is very important, because they will be the one to able to speak better on it because they will be the frequent users.’ (Participant 9, Botswana, Man, Physical disability)

*Terminology* used seemed easier to follow than in earlier versions because much fewer challenges were identified in this area than with the pilot study (Visagie et al. 2018).

‘It easy and understandable.’ (Participant 11, Botswana, Man, Albinism)

Two participants still mentioned that ‘eating and drinking’ can be confusing as people might think it refers to eating places instead of adaptive eating and drinking devices.

‘When entering data into the app we might see so many information like Eating and Drinking, which does not mean we are going to find food and drinks but all the facilities (devices) that can help persons with disabilities to eat and drink if in need of that.’ (Participant 3, Lesotho, Woman, Intellectual disability)

The *inclusiveness of the app* was criticised. Participants felt that people who are not *fluent in English* were excluded.

‘Language barrier when using the app, I sometimes need someone who understand the English language to help me.’ (Participant 15, Lesotho, Man, Physical disability)

They suggested that the app is translated into African languages.

‘The information and the questions are fair to understand but since many Malawians understand their mother tongue language if the app should translated into Chichewa the language every Malawian understands clearly.’ (Participant 6, Malawi, Woman, Disability)

‘We think we have to add our language so that we can use it easily. We propose that google must translate English to African languages.’ (Participant 19, Lesotho, Man, Hearing impairment)

Those with *visual impairments* were also excluded.

‘However, as a person with visual impairment, like when it comes to the device, like the phone which accepts this application is not user friendly to me hence it poses a challenge that I can be able to use because these phones are using touch screen they don’t have keys that I will be able to use. Then, what makes it more difficult is because icons and dialogue boxes, these are graphics hence I will not be able to see them so I can click maybe on delete when I am not supposed to delete. If it can be made easier even the phones that have keys can be able to accept the product itself. Then like it was said on these other icons where you just have a person on a wheelchair without the wordings it becomes a challenge even when you’re able to use a computer, as persons with visual impairment we use key strokes or shortcut keys. So for me to locate that particular picture where the software maybe which I may be using on that particular phone cannot even read or describe the picture which is appearing on that particular icon, it will not be user friendly but it’s a very good product what we need to have is it being made more accessible that it breaks the barriers which are there.’ (Participant 17, Zambia, Man, Sensory disability)

Some participants felt that not all could afford *Android phones* and that a similar app for phones with keypads and/or computers will enhance access. This will also allow better access for persons with visual impairments.

‘Looking at our status in Botswana mostly for people living with disabilities, most of us are not working, most of us are not well educated, meaning most of us don’t have smartphones, if you looking people living in rural areas they only have simple phones which is not compatible with Commcare [*platform of AT-Info-Map app*].’ (Participant 11, Botswana, Man, Supplier of mobility devices)

‘Malawi is a developing country and many people don’t own smart phones and this is a big challenge for the app to work in the country.’ (Participant 4, Malawi, Man, Disability)

It was further suggested that *mobile service companies* could help by providing smart phones in exchange for marketing.

‘How are you working with for instance the mobile service providers and the shops that are selling these phones, maybe we can have some of the phones that can accommodate this same software and make their phones advertised on this same platform so that we can know that maybe its Mobile City that is selling a mobile phone of this nature, we can rush there as quick as possible so that we can be able to access this application because without a phone I will not access these services easily. So if those people who are this particular business are informed and educated about this product they will also want to market to us and go into a program where we can maybe make it flexible to those who can be able to pay for such kind of phones.’ (Participant 17, Zambia, Man, Sensory disability)

The fact that the app can be used offline once downloaded was seen as a big advantage and cost saving.

‘Something I like the most about this application is you can use it offline most of the times once installed. All you need is interval updates maybe every after a week or two just to be updated with the new information being uploaded.’ (Participant 19, Zambia, Man, Intern at Zambian Federation for the Disabled)

However, some still felt that even the *data* needed to download the app might be too much.

‘Data in Botswana is very expensive, it will be a very big challenge and it will be problematic in using the app.’ (Participant 7, Botswana, Woman, Social worker)

‘On the issue of data consumption, app developers should think outside the box in coming up with possible solutions for the app to be downloaded for free. Many Malawians are poor and earn as low as less than a dollar a day and can’t afford buying a bundle for the app download.’ (Participant 7, Malawi, Man, Supplier of AT)

## Discussion

Findings showed an overall positive response to the AT-Info-Map app, and usefulness for AT users, suppliers and NGOs. However, improvements to the app design and continual system maintenance are required to ensure broad uptake and sustainability.

Similar to previous studies (Allsop et al. 2018; Vesel et al. 2015; Watkins et al. 2018), focus group participants identified current and potential future challenges with the AT-Info-Map app, and barriers to using mobile applications in southern Africa like the cost of smart phones and data. App challenges, such as difficulty logging in, clarity of some AT icons, lack of full accessibility by people with visual impairments and lack of translated versions in multiple languages, were consistent with the findings in the evaluation of the pilot phase of the project (Visagie et al. 2018). While many issues identified during the pilot phase were addressed in the final version of the app, these findings showed that improvements were not sufficient to ameliorate usability and technical issues for all users.

Improvements implemented by SAFOD included (1) developing a Portuguese version of the app; (2) testing with TalkBack, a built in screen reader on Android phones, to ensure data entry and data searching were accessible to persons with visual impairments; (3) reducing data demand of the app and (4) creating training resources such as manuals and online videos to assist app access and usage, and educate the general public about AT. Shortcomings include that the app is not translated for any indigenous language within southern Africa, and that not all contents (i.e. icons) are fully accessible to people with visual impairments. In addition, some focus group participants pointed out they still experienced technical challenges such as installing and navigating the app, after having received in-person training on the app.

The cost of phones and data will continue to be a major challenge to broader uptake. The cost of data is indeed high in the study countries and southern Africa in general, varying from $6.6 (US) in Malawi to $12.6 (US) in Zambia for 1 GB (RAMP index 2017; https://businesstech.co.za/news/mobile/185941). Furthermore, in agreement with current findings, access to smart phones and stable Internet connections are not given in southern Africa (Opoku et al. 2017).

The two major interrelated concerns expressed by participants about the future of the AT-Info-Map app were ensuring accuracy of data and sustainability. Those concerns are not unique to this project, and ongoing funding is required to address these issues. The caution that information provided on the app must be accurate and regularly updated is an important one that must be heeded as Opoku et al. (2017) found accuracy of information to tie in with sustainability of apps. Currently, verifying the correctness of data on the app is achieved through contacting suppliers or service organisations via phone, email or in person. Verifying records is a time-intensive process that will be costly in the long term. Therefore, SAFOD is exploring ways to incentivise suppliers and organisations to update their own information annually. Sustainability of the app and web-based system also involves ongoing maintenance and possible further iterations in response to a wide range of user needs.

Some focus group participants had the expectation that specific products could be sourced directly through the app (i.e. the reference to finding products, and the suggestions that pictures and prices are added). Currently, the app cannot support this level of detailed information. It is not clear if these participants did not understand the type of information available in the app, or if their feedback solely aimed to inform future improvements. Another expectation of participants was that the app will provide AT suppliers with information on what products are in demand and that this aggregated information will increase sales of AT.

However, users of AT often do not purchase directly from suppliers because AT services or rehabilitation specialists are required to access many types of AT (e.g. hearing aids, wheelchairs, prosthetics) (Smith et al. 2018). Furthermore, there is an overall lack of funding for AT in southern Africa, through inadequate public financing and inability of users to pay. Lack of financial resources among people with disabilities in developing countries has been well documented (Hanass-Hancock et al. 2017; Mitra, Posarac & Vick 2011), and the limited AT available is often subsidised through governments and NGOs (Visagie et al. 2016). For these reasons, the estimates of AT demand generated through the app will not automatically translate into increased sales.

Despite the challenges and limitations with the current app, many focus group participants found value in the data collected and were enthusiastic about the potential for this information system to facilitate networking and growth in the AT sector in southern Africa. Participant feedback validated the first objective of the AT-Info-Map – to assist users and providers of AT to locate AT. For example, some participants found the geographical mapping feature useful in saving time and money in locating AT suppliers of closer proximity. The second objective – to show gaps in the availability of AT in southern Africa – was mentioned in focus group discussions in terms of assessing unmet demand for suppliers, but not as a tool for informing advocacy or policy-making.

As the first attempt to create a regional information system that compiles data on AT suppliers and disability service organisation, the enthusiasm may also reflect approval of, and the broader vision of, the AT-Info-Map to increase access to AT through mapping availability. However, the app itself has much room for improvement, and ongoing funding is required to ensure sustainability of the system. It is worth noting that similar country-level or regional AT data systems, such as Assistive Technology Data-Denmark (https://hmi-basen.dk/en/) or European Assistive Technology Information Network (http://www.eastin.eu/en/searches/products/index), are government funded and integrated into the public AT systems. These more developed and publicly supported systems offer more detailed AT information such as product specifications and pricing, a few of the features desired by participants. While SAFOD and their affiliates have conducted the time-intensive task of compiling AT supplier information within a 10-country region into one searchable platform, the information system is unlikely to achieve its full potential without public investment from national governments and/or the Southern African Development Community (SADC) (Vesel et al. 2015).

### Limitations

The findings presented in this article report on the implementation of the AT-Info-Map app. While the app developers and authors strived to implement ethical and sound methodological processes during monitoring, some limitations are evident. Focus group participants were conveniently sampled rather than purposively. Data were collected from several facilitators by persons who were SAFOD employees, with different professional backgrounds. These factors could have led to bias in findings as a convenient sample negatively impacts credibility of findings and participants might have been aware of SAFOD’s involvement with the app, and thus less willing to share negative experiences or thoughts. Including different facilitators could have led to a variety in the quality of data between countries, and with that an unequal representation of countries in the narrative examples. Although data from South Africa had to be excluded, the transcripts from South Africa showed no different themes to those identified in the other countries. However, by giving country branches of SAFOD control over the data collection process in their respective countries, they were affirmed and skills were developed among their members.

## Conclusion and way forward

The information on AT as presented on the app is only a partial solution to AT access in southern Africa, even if all technical problems were addressed. Lack of policy and poor policy implementation, lack of AT awareness, supply chain challenges, lack of trained service providers and lack of money all hamper access to AT in the setting. At the same time, as the first attempt to map availability of AT across 10 countries it is an important step in the right direction. Looking forward, participants encouraged SAFOD to sustain the AT-Info-Map data, continually improve the app and ensure that data are accurate and up-to-date, a task that might not be possible without government support.

While SAFOD was able to address some technical and programmatic challenges through app modifications and training, the app challenges motivated SAFOD to develop a web-based system that will complement the app, and re-develop the app on a more user-friendly platform. The web-based system allows for more detailed product-level information, greater access for persons with visual impairments and the option to use Google Translate (https://assistivetechmap.org/). Both the web system and the new app will reduce many barriers to access and use, but will remain out of reach to those without Internet access. To mitigate marginalisation of those not formally educated and people residing in rural areas, regular outreach programmes where organisations for persons with disabilities gather people and share information from the app are recommended. Both outreach programmes and the web-based system must be evaluated at a later stage.
